# Collagen-Coated Poly(lactide-*co*-glycolide)/Hydroxyapatite Scaffold Incorporated with DGEA Peptide for Synergistic Repair of Skull Defect

**DOI:** 10.3390/polym10020109

**Published:** 2018-01-24

**Authors:** Ming Bi, Hui Han, Shujun Dong, Ying Zhang, Weiguo Xu, Bitao Zhu, Jingyun Wang, Yanmin Zhou, Jianxun Ding

**Affiliations:** 1 Department of General Dentistry, School and Hospital of Stomatology, Jilin University, Changchun 130021, China; biming11@mails.jlu.edu.cn (M.B.); jlccjingyun@sina.com (J.W.); 2 Department of Thyroid Surgery, The First Hospital of Jilin University, Changchun 130021, China; hh198404@hotmail.com; 3 VIP Integrated Department, School and Hospital of Stomatology, Jilin University, Changchun 130021, China; dsj@jlu.edu.cn; 4 Department of Orthopedics, Zhongshan Hospital Affiliated to Xiamen University, Xiamin 361004, China; 5 Key Laboratory of Polymer Ecomaterials, Changchun Institute of Applied Chemistry, Chinese Academy of Sciences, Changchun 130022, China; wgxu@ciac.ac.cn (W.X.); btzhu@ciac.ac.cn (B.Z.)

**Keywords:** porous scaffold, collagen coating, bioactive peptide, skull defect repair, tissue engineering

## Abstract

The treatment of large-area bone defects remains a challenge; however, various strategies have been developed to improve the performances of scaffolds in bone tissue engineering. In this study, poly(lactide-*co*-glycolide)/hydroxyapatite (PLGA/HA) scaffold was coated with Asp-Gly-Glu-Ala (DGEA)-incorporated collagen for the repair of rat skull defect. Our results indicated that the mechanical strength and hydrophilicity of the PLGA/HA scaffold were clearly improved and conducive to cell adhesion and proliferation. The collagen-coated scaffold with DGEA significantly promoted the repair of skull defect. These findings indicated that a combination of collagen coating and DGEA improved scaffold properties for bone regeneration, thereby providing a new potential strategy for scaffold design.

## 1. Introduction

Partial or large bone resection is needed for the treatment of bone tissue lesions caused by trauma, tumors, and other diseases. Postoperative bone recovery is often poor, resulting in delayed healing, nonunion, and poor bone regeneration, which poses great challenges in the fields of orthopedics and stomatology [1]. Autologous bone grafting is the “gold standard” for the treatment of large-area bone defects; however, limited source material in donors, complicated shapes and sizes of damaged bones, and the creation of bone lesions at extraction sites severely limit the extensive use of bone grafting in the clinic. Bone tissue engineering, an important field of tissue engineering, provides good prospects for the replacement and regeneration of bone tissue. Scaffolds, one of the three elements of tissue engineering, play an important role in bone repair [2]. The bone tissue engineering scaffolds currently used by researchers mainly include biodegradable polymer materials, titanium alloys, and calcium phosphate [3]. Especially for the polymer materials, their low biological activity, poor tissue-cell compatibility, and slow or nonexistent biodegradation lead to poor therapeutic efficacy, requiring these scaffolds to be often removed in a second surgery, which places great physical and financial burdens on patients [4]. Furthermore, the transplantation of bone tissue engineering scaffolds is a very complex and multifactorial process that involves the interaction and regulation of scaffolds, cells, and molecules at different levels [5]. Therefore, the design and optimization of bone tissue engineering scaffolds has important clinical significance.

Bioactive substance coating is an important method used to improve the physical and biological properties of bone tissue engineering scaffolds [6,7]; however, insufficient biocompatibility of the coating matrix and limited sources greatly limit its application. Collagen is an important organic compound in the human body, accounting for about 80% by volume of bone tissue, and plays an irreplaceable role in the construction and maintenance of bone histomorphology [8]. The interaction between cells and collagen can promote osteogenic differentiation of bone marrow stem cells, and collagen can also be combined with many biological molecules and cytokines to regulate and promote the growth and differentiation of stem cells [9]. Compared with growth factors, the short peptides are more stable and low-cost. Some short peptides, such as FHRRIKA (Phe-His-Arg-Iso-Lys-Ala), KRSR (Lys-Arg-Ser-Arg), and DGEA (Asp-Gly-Glu-Ala), have been reported to enhance cell adhesion and matrix mineralization [10]. DGEA is a collagen-derived short peptide that plays a significant role in osteoinduction. Because bone regeneration is a long-term, slow process, DGEA was expected to act as a sustained release osteoinductive factor that can continue to play a role in bone repair.

**Scheme 1:**
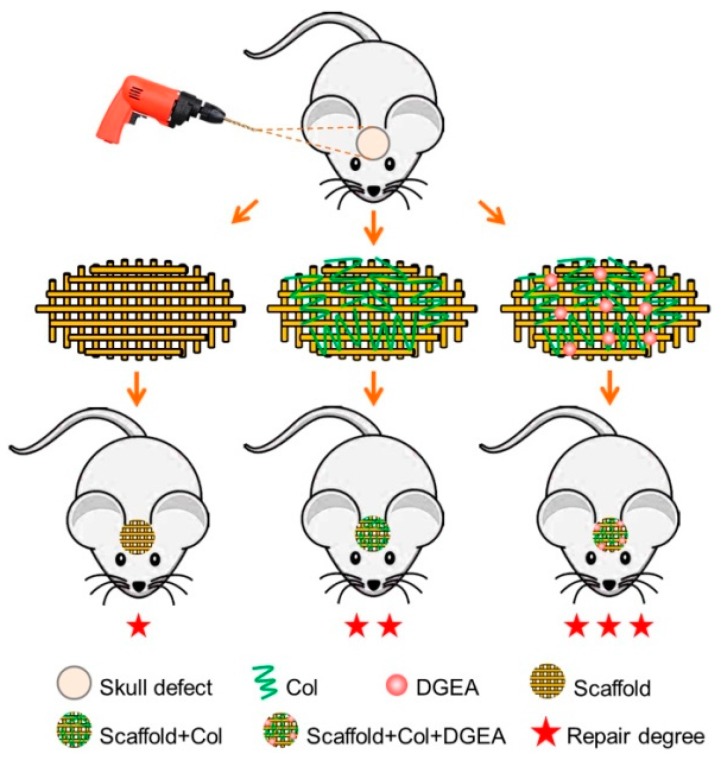
Schematic illustration of model establishment, defect repair with scaffolds, and effect evaluation.

Therefore, in this study, poly(lactide-*co*-glycolide)/hydroxyapatite (PLGA/HA) scaffold was coated with DGEA-incorporated type I collagen (Col I) to optimize the performances of the scaffold (Scaffold+Col+DGEA) (Scheme 1). Based on its good biological activity, collagen can promote cell proliferation and adhesion by binding to α_2_β_1_ integrin on the cell surface, resulting in formation of a three-dimensional (3D) structure due to the molecular chains in collagen and leading to the slow release of DGEA. To verify these effects, a rat model of skull defect was designed and constructed. Using micro-computed tomography (micro-CT) and immunohistochemistry, Scaffold+Col+DGEA was demonstrated to significantly promote the repair of skull defects at 12 weeks after scaffold implantation.

## 2. Materials and Methods

### 2.1. Reagents and Materials

Collagen and 4′,6-diamidino-2-phenylindole (DAPI) were purchased from Sigma-Aldrich (Shanghai, China); short-peptide DGEA from ChinaPeptides Co., Ltd. (Shanghai, China); PLGA/HA scaffolds from SinoBiomaterials Co., Ltd. (Changchun, China); Trizol, the reverse transcription kit, and the real-time reverse transcription polymerase chain reaction (RT-PCR) kit from Vazyme Biotech (Nanjing, China).

The field-emission scanning electron microscope (FESEM, XL30ESEM-FEG) was purchased from FEL (Amstelveen, The Netherlands); the electronic universal testing machine from Instron Co. (High Wycombe, UK); the drop analyzer from Kruss Co. (Hamburg, Germany); the micro-CT from Bruker Co. (Ettlingen, Germany); the T100 thermal cycler from Bio-Rad (Hercules, CA, USA); the Milli-Q Plus ultrapure water system from Millipore (Billerica, MA, USA); and the primary antibody, osteocalcin (OCN), and secondary antibody, rabbit anti-rat, from Bioss Co. (Beijing, China).

### 2.2. Preparation of Coated Scaffolds

Collagen was fully dissolved in phosphate-buffered saline (PBS; 0.01 M; pH 7.4) and formulated into collagen solution at a concentration of 10.0 mg·mL^−1^. Each PLGA/HA scaffold was made into a circular scaffold with a diameter of 5 mm and a thickness of 2 mm, and placed in a safety bottle, which was evacuated, thereby creating negative pressure inside the scaffold. When collagen solution was injected, it completely immersed the scaffold under negative pressure. The scaffold was shaken on a shaking bed 50 times min^−1^ for 10 min, thereby allowing the collagen to be uniformly coated on the pore surface. After freeze-drying, the scaffold was stored for further use. Scaffold+Col+DGEA was created using the same method, although the collagen solution was replaced with a DGEA-incorporated collagen solution. The concentration of DGEA in collagen was 1.0 mg·mL^−1^.

### 2.3. Characterization of Coated Scaffolds

#### 2.3.1. Morphological Observation

The scaffold material was broken by liquid nitrogen, and the scaffold cross-section was sputtered with gold using a rotating sputtering machine. The pore size and microstructure of scaffold materials were observed using a FESEM.

#### 2.3.2. Mechanical-Property Testing

The scaffold material was cut into 10 × 10 × 10-mm blocks, and a compression test was performed with an electronic Universal Testing Machine (Instron 1121, Canton, MA, USA) at a rate of 2 mm·min^−1^. The test standard was accorded to GB/T 1041-1992, China. 10 samples for each group have been tested. The maximum compressive strength was separately recorded when the scaffold material was deformed to 10%, 25%, and 50%.

#### 2.3.3. In Vitro Degradation Experiment

The scaffold material was cut into 10 × 10 × 10-mm blocks, which were completely immersed in 20.0 mL of PBS at pH 7.4 and placed in a 37 °C thermostat container. PBS was replaced every two days, and water in the scaffold was simultaneously removed with filter paper. Weight changes in the scaffold materials were recorded.

#### 2.3.4. Hydrophilicity Detection

The water contact angle (WCA) of the scaffold was detected with a flat surface. When the droplet contacted the surface of the scaffold, its final shape was dependent upon the internal cohesion and the adhesive force on the surface of the scaffold. Specifically, 20.0 μL of liquid was dropped onto the surface of the scaffold, and the contact angle was photographed and recorded with a drop analyzer.

### 2.4. In Vivo Animal Experiments

Healthy male, specific pathogen free rats aged 7 weeks and weighing ~300 g were provided by the Beijing Vital River Laboratory Animal Technology Co., Ltd. (Beijing, China). Animal use was in line with the guidelines of the National Institutes of Health (NIH) for animal studies (Principles for Feeding and Using Laboratory Animals) and approved by the Animal Ethics Committee of Jilin University (Approval No.: AEC-2017-01, Approval Date: 12 January 2017).

Ozonization was performed for 2 h before scaffold implantation, followed by ventilation in a super clean bench for 12 h. The rats were randomly assigned to four groups (*n* = 12/group): blank (Control) group; PLGA/HA scaffold (Scaffold) group; PLGA/HA+collagen (Scaffold+Col) group; and PLGA/HA+collagen+DGEA (Scaffold+Col+DGEA) group. All rats were sacrificed by anesthesia at 6 or 12 weeks after implantation.

#### 2.4.1. Establishment of Animal Models

The rats were anesthetized by intraperitoneal injection of 0.3 mL/100 g 10% chloral hydrate (CHL). After shaving, routine disinfection, and spreading the sheet, an incision was made along the midline of the skull. The skin, soft tissue, and periosteum were dissociated layer by layer to expose the flattest part of the rat parietal bone. Taking the sagittal suture as the midline, a circular defect 5 mm in diameter was drilled at the center of the parietal bone with a trephine. Different scaffolds were implanted in the corresponding groups, with the blank group not receiving any scaffold. Ultimately, the incision was sutured layer by layer. Animal activities, diet, and mental status, as well as the incisions, were observed after surgery. Penicillin (400,000 U) was injected intramuscularly into limbs daily for 1 week.

#### 2.4.2. Micro-CT Detection

Rat skull samples were placed on the sample table, photographed, and analyzed using micro-CT. X-ray scanning was performed at a resolution of 1000 k and a thickness of 0.1 m per layer. Parameters of tomographic images were corrected using CTAn analysis software (Bruker Co.) and reconstructed, followed by analysis of relevant bone parameters. Meanwhile, bone mineral density (BMD) was tested compared to normal bone tissue through micro-CT.

#### 2.4.3. RNA Extraction and RT-PCR Assays

New skull tissue at the defect site was isolated and cut with RNase-free eye scissors. These tissue pieces were frozen quickly in liquid nitrogen, triturated, and treated with 1.0 mL of Trizol reagent and 0.2 mL of chloroform. After shaking for 5 min, all specimens were centrifuged for 15 min at 10,000 *g* and at 2 to 8 °C. The upper aqueous phase was incubated with 0.5 mL of isopropanol at room temperature for 5 min and centrifuged at 10,000 *g* and at 2 to 8 °C for 10 min. After supernatant removal, the RNA precipitate was washed with 75% ethanol and centrifuged at 7500 *g* and at 2 to 8 °C for 5 min, followed by discarding of the supernatant. RT-PCR was conducted in strict accordance with the manufacturer instructions. Gene expression was determined using immunofluorescence staining.

#### 2.4.4. Immunofluorescence Staining

The rats were sacrificed at 6 and 12 weeks. Skulls were fixed in 4% (*w*/*v*) paraformaldehyde/PBS, placed in a 37 °C incubator, and decalcified with ethylenediaminetetraacetic acid (EDTA) for 30 days. The decalcifying fluid was replaced twice weekly. Specimens were embedded in paraffin and sliced into 5-μm thick sections in the Department of Pathology, The First Hospital, Jilin University (Changchun, China). Sections were heated in an oven at 60 °C for 2 h. After dewaxing with xylene, the sections were washed with a graded alcohol series. Antigen was retrieved with 0.01 M citric acid/sodium citrate (CA/NaC) buffer solution (pH 6.0), followed by three washes with PBS (3–5 min each). The sections were then incubated with goat serum in a wet box at 37 °C for 20 min, followed by the primary antibody at 4 °C overnight and finally rewarmed in a 37 °C incubator for 10 min. After three washes with PBS (3–5 min each), the sections were incubated with the secondary antibody at 37 °C for 40 min, washed three times with PBS (3–5 min each), stained with DAPI (2–3 min), washed three times with PBS (3–5 min each), mounted with glycerol, and observed with an LSM780 confocal laser scanning microscope (CLSM; Carl Zeiss AG, Oberkochen, Germany).

### 2.5. Statistical Analyses

All data were processed and analyzed with GraphPad Prism 7.0 (GraphPad Software, La Jolla, CA, USA). All experiments were repeated at least three times and all data are expressed as means ± standard deviation. Mean data were compared using Student’s *t*-test, and a * *p* < 0.05 was considered to indicate statistically significant differences. ^#^ *p* < 0.01 was considered to indicate distinct significance. ^&^ *p* < 0.001 was considered to indicate very distinct significance.

## 3. Results and Discussion

### 3.1. Characterization of Coated Scaffolds

**Figure 1:**
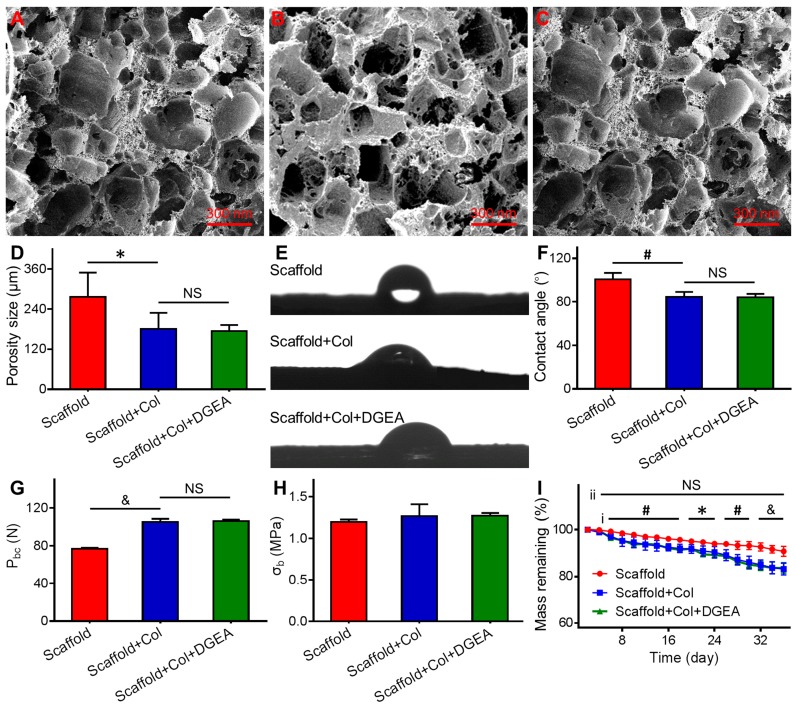
Morphologies, mechanical strength, and degradation. (**A**–**C**) Morphologies; (**D**) porosity sizes; (**E**) morphologies of contact angle measurement; and (**F**) contact angles of Scaffold, Scaffold+Col, and Scaffold+Col+DGEA; (**G**) Compressive loads; (**H**) compressive tests; and (**I**) degradation rates of Scaffold, Scaffold+Col, and Scaffold+Col+DGEA (*n* = 3; * *p* < 0.05, ^#^ *p* < 0.01, ^&^ *p* < 0.001; i means Scaffold group vs. Scaffold+Col group; ii means Scaffold+Col group vs. Scaffold+Col+DGEA group).

Scanning electron microscopy revealed that the pore size of the scaffold was uniform, some pores were perforated, and the inner wall was smooth (Figure 1A), which contributed to cell adhesion and substance exchange [11,12]. As displayed in Figure 1A–D, after collagen coating, the pore size changed from 276 to 180 μm. A previous study showed that a pore size of 150 to 500 μm was conducive to mineralization of the bone matrix [13]. Our results indicated a coarser inner wall of the coated scaffold, which would be more conducive to cell adhesion (Figure 1B,C).

Collagen is the main organic component of bone tissue and accounts for 90% of extracellular matrix proteins, with the most abundant type of collagen being Col I (~97%) [14]. Collagen interacts with many active molecules and growth factors to promote the proliferation and differentiation of cells. Geissler et al. found that collagen-coated metal and artificial bone effectively improved scaffold performance and promoted bone regeneration [15,16].

PLGA is a common biodegradable polymer and has a wide range of applications in the field of biomedicine [17,18]. As the primary bone tissue engineering scaffold, PLGA has been used as a bone substitute in the clinic [19]; however, its use presents some problems, including a lack of hydrophilicity. Moreover, acidic substances, such as lactic acid and glycolic acid, can be produced during PLGA degradation, as these acidic substances are not conducive to cell adhesion or growth [20]. When WCA is >90°, the surface of the material is hydrophobic, and at <90°, it is hydrophilic. As shown in Figure 1E,F, the WCA was reduced from 100.8° ± 5.7° to 84.8° ± 5.7°or 84.0° ± 3.2° after collagen coating, suggesting that the coating effectively improved scaffold hydrophilicity.

The presence of collagen in the scaffold clearly improved its mechanical properties. The maximum compression load increased from 76 to 105 or 106 N (Figure 1G), but the compressive strength had no obvious change (Figure 1H). Strength is highly significant for bone repair scaffolds used in bone regeneration, with higher-strength materials more conducive to stem cell differentiation into osteoblasts [21,22]. Therefore, high-strength bone tissue engineering scaffolds play a role in bone induction, provide a good platform for cell growth and differentiation, and are viable in the treatment of weight-bearing bone defects.

The collagen coating also influenced scaffold degradation. The quality of scaffolds on the first day was used as a baseline to record the change in scaffold weight. As shown in the in vitro degradation assay (Figure 1I), the same change of quality was accompanied by a faster degradation rate in the Scaffold+Col group relative to that in the Scaffold group. At 5 weeks, the mass remaining percentages were 83.2% ± 2.5% and 84.0% ± 2.3% in the Scaffold+Col and Scaffold+Col+DGEA groups, respectively, but 90.8% ± 2.2% in the Scaffold group. During the scaffold degradation process, the pore became much larger, and the surface became much rougher. We expected that Scaffold+Col would be conducive to cell adhesion and proliferation after implantation. On the one hand, adherent macrophages can accelerate scaffold degradation with phagocytosis; however, adherent osteoclasts play an irreplaceable role in osteogenesis [23]. Furthermore, DGEA, which exhibits osteogenic effect, was mixed with collagen. All in all, as shown in Figure 1, Scaffold+Col+DGEA showed the similar properties as Scaffold+Col. Therefore, the gradual degradation of collagen would be expected to achieve the slow release of DGEA and sustain osteogenesis.

### 3.2. In Vivo Efficacy Verification

To further verify the effect of the collagen coating onto scaffold on bone repair, different repair scaffolds (i.e., Scaffold, Scaffold+Col, and Scaffold+Col+DGEA) were implanted into skull defect sites in rats. At 6 and 12 weeks after implantation, the rats were euthanized and subjected to related experiments.

**Figure 2:**
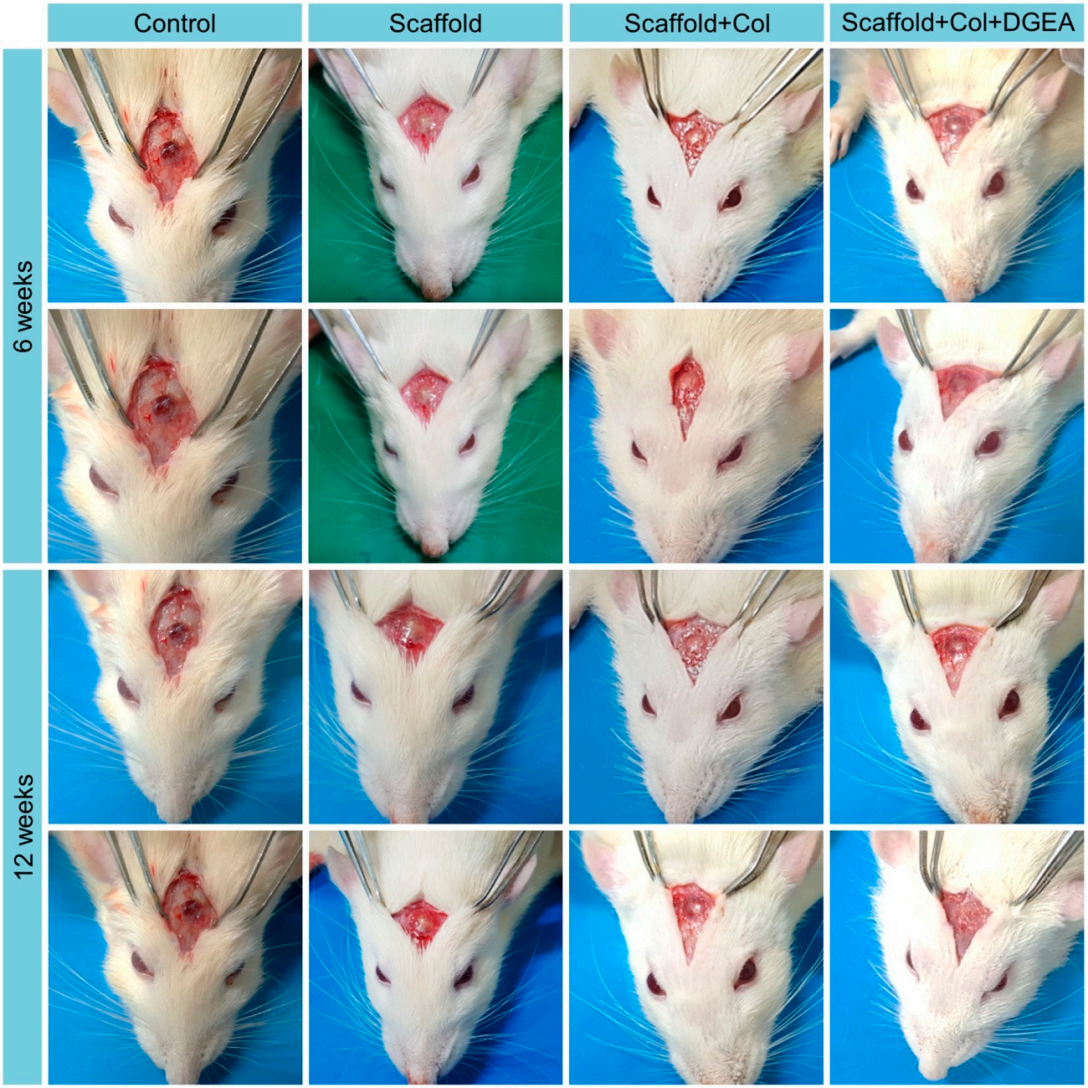
Gross morphologies of skull defect sites at 6 and 12 weeks post-implantation.

Generally, bone regeneration can be divided into three stages: hematoma formation, mechanization, and braided bone formation; callus formation and mature bone increase; and callus modelling [24,25]. At 6 and 12 weeks post-implantation, the site of bone defects was photographed. In the control group, defect sites were still in the stage of fiber repair, and no dense tissue had appeared (Figure 2). In the Scaffold and Scaffold+Col groups, new tissue was visible in the defect areas, and the defect areas had become small. Due to the sustained release of DGEA, many new tissues were found at the defect sites in the Scaffold+Col+DGEA group, indicating that these scaffolds exhibited the best repair effects on bone defects.

**Figure 3:**
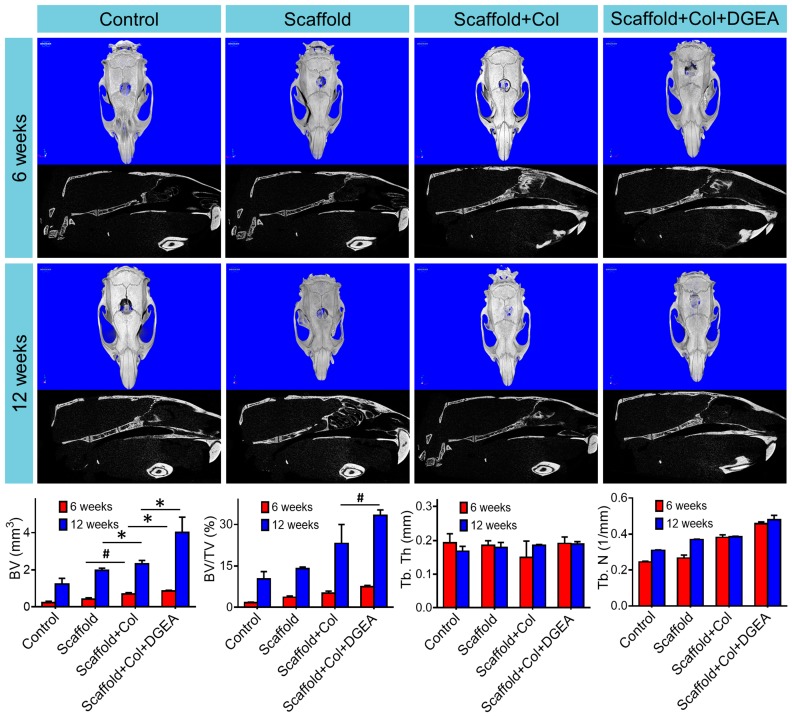
3D reconstruction of skull bones using micro-CT and semi-quantitative analyses of skull defect sites (*n* = 5; * *p* < 0.05, ^#^ *p* < 0.01).

Rat skulls were removed and analyzed by micro-CT scanning and underwent 3D reconstruction and bone-related parameter measurement. As displayed in Figure 3, 3D reconstruction images revealed that skull defects were obvious in each group at 6 weeks. In the control group, no new bone tissue was found at the defect sites. In the other scaffold groups, new bone tissues were visible at the defect sites; however, no significant differences were found among these three groups. At 12 weeks, skull defects had become small in the scaffold groups. The repair effect was clearest in the Scaffold+Col+DGEA group, with almost complete healing of the defects. The defect sites observed in the 3D reconstruction images were considered regions of interest (ROIs), and data were further processed and analyzed using CTAn software (Bruker Co.; Figure 3). Bone volume and bone volume/total volume at the defect sites in each group were significantly higher at 12 weeks than those at 6 weeks. At 12 weeks, bone volumes in the ROIs of rats in the Scaffold+Col+DGEA group were 2.0 and 1.7 times larger than those in the Scaffold and Scaffold+Col groups, respectively. The bone volume/total volume in the ROIs in the Scaffold+Col+DGEA group was 2.4 and 1.4 times larger than that in the Scaffold and Scaffold+Col groups, respectively. No significant difference in trabecular thickness or number in ROIs of the rat skulls was determined among the groups. As shown in Figure S1, BMD, 0.88-fold relative to normal bone tissue, was highest among each group.

**Figure 4:**
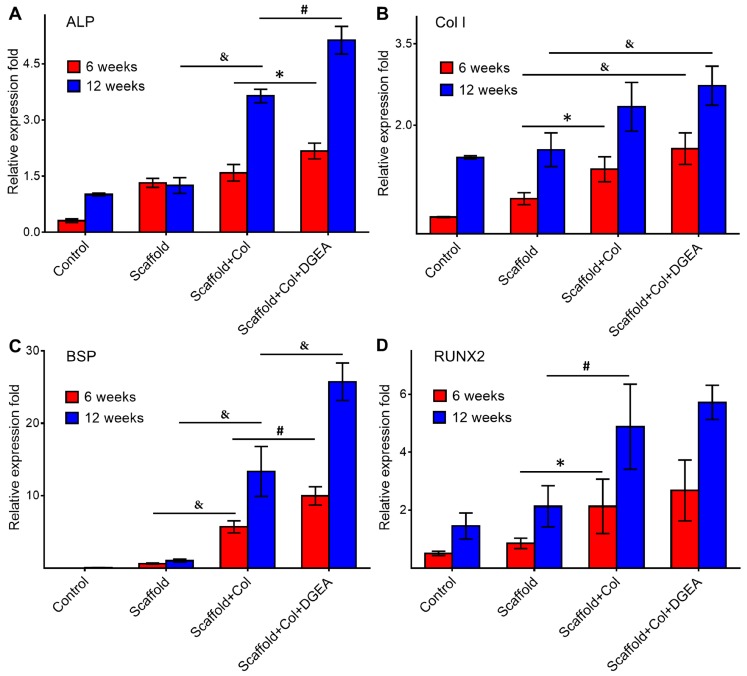
Expression of (**A**) ALP; (**B**) Col I; (**C**) BSP; and (**D**) RUNX2 mRNA in skull defect sites as detected by RT-PCR at 12 weeks post-implantation (*n* = 5; * *p* < 0.05, ^#^ *p* < 0.01, ^&^ *p* < 0.001).

In this study, total RNA was extracted from the skull defect sites of rats. After reverse transcription, RT-PCR was used to measure the expression of alkaline phosphatase (ALP), collagen I (Col I), bone sialoprotein (BSP), and runt-related transcription factor 2 (RUNX2) mRNA. As shown in Figure 4A, at 6 weeks, ALP expression relative to that of β-actin was lowest in the Control group by 0.31-fold, and there was no significant difference in relative ALP expression between the Scaffold and Scaffold+Col groups. Additionally, relative ALP expression was highest in the Scaffold+Col+DGEA group, 1.57 times relative to that of β-actin. At 12 weeks, relative ALP expression was significantly different among groups, with increases by 1.25, 3.64, and 5.13 times in the Scaffold, Scaffold+Col, and Scaffold+Col+DGEA groups, respectively, relative to β-actin in these groups. These results suggested that the collagen coating and sustained release of DGEA promoted the in-growth of related cells, increased ALP expression, and promoted osteogenesis. At 12 weeks, scaffolds with collagen or DGEA effectively promoted Col I expression, which was highest in the Scaffold+Col+DGEA group, 2.73 times relative to β-actin (Figure 4B). Highly expressed Col I produced more bone matrix, which contributed to the deposition of calcium salts and cell adhesion. BSP is strongly associated with osteogenic differentiation, and BSP expression showed a trend similar to that of Col I (Figure 4C). Relative BSP expression was nearly undetectable in the Control and Scaffold groups; however, over 6 to 12 weeks, relative BSP expression increased in the Scaffold+Col and Scaffold+Col+DGEA groups, with the highest relative BSP expression observed in the Scaffold+Col+DGEA group at 12 weeks, 25.73 times relative to β-actin. RUNX2 expression was strongly associated with osteoblast differentiation, and relative RUNX2 expression in each group was significantly higher at 12 weeks than at 6 weeks, 2.13 and 4.88 times in the Scaffold and Scaffold+Col groups, respectively (Figure 4D). However, no significant difference was observed between RUNX2 expression in the Scaffold+Col and Scaffold+Col+DGEA groups. These results suggested that Scaffold+Col+DGEA group showed the best reparative effect on skull defects. Our findings indicated that gradually degrading collagen and sustained release of short-peptide DGEA played important roles in skull repair.

ALP is a marker of early-stage osteoblast differentiation and can provide necessary phosphate for hydroxyapatite, thereby playing an important role in bone formation and mineralization. In the clinic, ALP measurement is often used to understand the bone metabolism of patients. Col I is a scaffold for calcium deposition and cell adhesion, and accounts for ~90% of the organic bone matrix. In the proliferation phase of osteoblasts, the number of osteoblasts increases, and Col I is synthesized and secreted, gradually mineralizing to form bone nodules. Therefore, Col I is of great importance to bone growth. BSP is a marker protein associated with the late stage of osteoblast differentiation and involved in signal recognition, cell adhesion, and migration. RUNX2, a member of the RUNX transcription factor family, is a target of the bone morphogenetic protein (BMP) signaling pathway and important in bone regeneration induced by BMPs. RUNX2 promotes BMP2-induced differentiation of mesenchymal stem cells (MSCs) into osteoblasts. Therefore, bone-specific RUNX2 is considered a specific marker for bone formation and mineralization.

**Figure 5:**
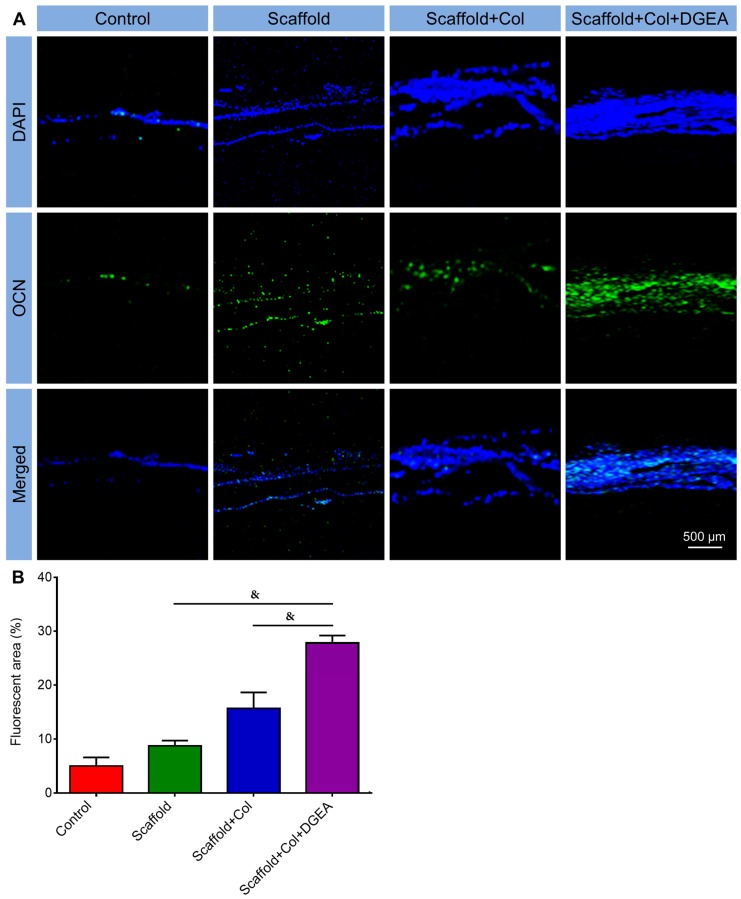
(**A**) Immunohistochemical and (**B**) semi-quantitative analyses of skull defect sites at 12 weeks post-implantation (*n* = 3; ^&^ *p* < 0.001). The cell nucleus is blue labeled with DAPI, and OCN is green labeled with fluorescein isothiocyanate (FITC).

To further verify the reparative effect of the scaffolds on skull defects, the immunofluorescence staining of OCN was performed, which is strongly associated with osteoblast maturation. The strongest green fluorescence signal was observed at defect sites in the Scaffold+Col+DGEA group at 12 weeks (Figure 5A). Meanwhile, as shown in Figure 5B, the fluorescence area in the Scaffold+Col+DGEA group was 27.8%, which was 5.6 times larger than that in the control group. The above results confirmed that the collagen-coated scaffold loaded with DGEA promoted bone regeneration.

## 4. Conclusions

The collagen coating on PLGA/HA scaffold improved the performances and elevated the mechanical strength and hydrophilic properties to promote cell adhesion and proliferation. The constructed Scaffold+Col+DGEA persistently released DGEA during the repair of rat skull defects, thereby playing a sustained role in promoting osteogenesis. This composite-coating scaffold showed good bone-reparative effect and great clinical application potential.

## Supplementary Materials

The result about BMD of new born tissue in defect region is available online at http://www.mdpi.com/2073-4360/10/2/109/s1.
