# Supplementary material for: Collagen-Coated Poly(lactide-co-glycolide)/Hydroxyapatite Scaffold Incorporated with DGEA Peptide for Synergistic Repair of Skull Defect
Source: Polymers (Basel). 2018 Jan 24;10(2):109. doi: 10.3390/polym10020109 (PMC6414902; doi:10.3390/polym10020109)
Supplement: Supplementary file 1 [file polymers-10-00109-s001.docx]

Article

Collagen-Coated Poly(lactide-*co*-glycolide)/Hydroxyapatite Scaffold Incorporated with DGEA Peptide for Synergistic Repair of Skull Defect

Ming Bi ^1^, Hui Han ^2^, Shujun Dong ^3^, Ying Zhang ^4,^*, Weiguo Xu ^5^, Bitao Zhu ^5^, Jingyun Wang ^1^, Yanmin Zhou ^1,^* and Jianxun Ding ^5,^*

^1^ Department of General Dentistry, School and Hospital of Stomatology, Jilin University, Changchun 130021, P. R. China; [biming11@mails.jlu.edu.cn](mailto:biming11@mails.jlu.edu.cn) (M.B.); [jlccjingyun@sina.com](mailto:jlccjingyun@sina.com) (J.W.)

^2^ Department of Thyroid Surgery, The First Hospital of Jilin University, Changchun 130021, P. R. China; [hh198404@hotmail.com](mailto:hh198404@hotmail.com) (H.H.)

^3^ VIP Integrated Department, Stomatological Hospital, Jilin University, Changchun 130021, P. R. China; [dsj@jlu.edu.cn](mailto:dsj@jlu.edu.cn) (S.D.)

^4^ Department of Orthopedics, Zhongshan Hospital Affiliated to Xiamen University, Xiamin 361004, People's Republic of China

^5^ Key Laboratory of Polymer Ecomaterials, Changchun Institute of Applied Chemistry, Chinese Academy of Sciences, Changchun 130022, P. R. China; [wgxu@ciac.ac.cn](mailto:wgxu@ciac.ac.cn) (W.X.); [btzhu@ciac.ac.cn](mailto:btzhu@ciac.ac.cn) (B.Z.)

*Correspondence: [jxding@ciac.ac.cn](mailto:jxding@ciac.ac.cn) (J.D.); [zhouym62@126.com](mailto:zhouym62@126.com) (Y.Z.); [xmzhangying@163.com](#mailto:xmzhangying@163.com) (Y.Z.)


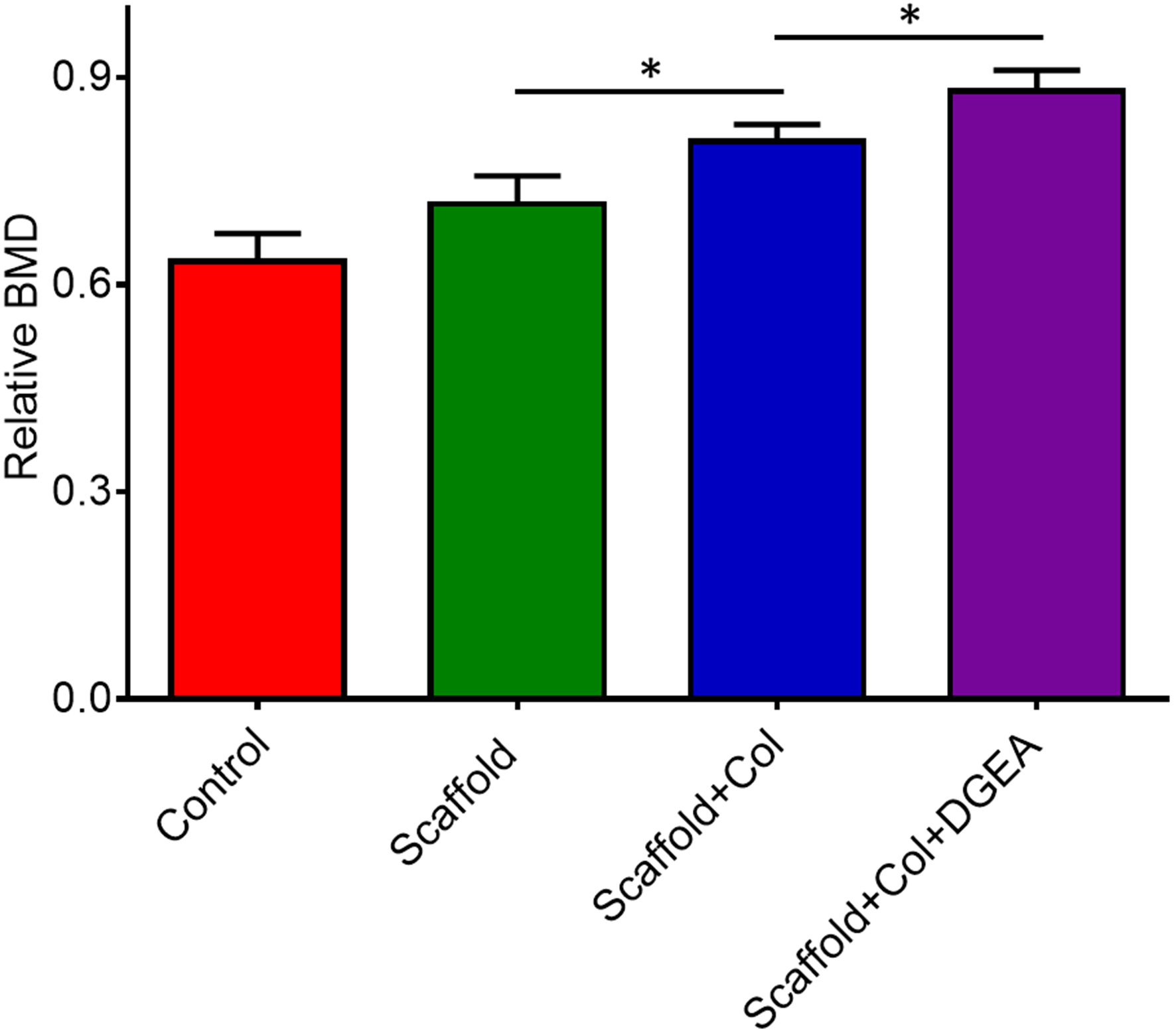


**Figure S1.** Bone mineral density (BMD) of new born tissue in defect region.
